# Middle and Long Latency Cutaneous Reflexes During the Stance Phase of Gait in Individuals with and Without Chronic Ankle Instability

**DOI:** 10.3390/brainsci14121225

**Published:** 2024-12-03

**Authors:** Leif P. Madsen, Annalee M. H. Friedman, Carrie L. Docherty, Koichi Kitano, David M. Koceja

**Affiliations:** 1 Department of Kinesiology, Indiana University, 1025 E 7th St, Bloomington, IN 47405, USA; cdochert@iu.edu (C.L.D.); kkitano@iu.edu (K.K.); koceja@iu.edu (D.M.K.); 2 Department of Applied Medicine and Rehabilitation, Indiana State University, 210 N 7th St, Terre Haute, IN 47809, USA; annalee.friedman@indstate.edu

**Keywords:** cutaneous reflexes, ankle instability, gait

## Abstract

Background/objectives: Lower limb cutaneous reflex amplitudes can modulate across gait, which helps humans adjust rhythmic motor outputs to maintain balance in an ever-changing environment. Preliminary evidence suggests people who suffer from repetitive ankle sprains and residual feelings of giving way demonstrate altered cutaneous reflex patterns in the gastrocnemius. However, before cutaneous reflex assessment can be implemented as a clinical outcome measure, there is a need to substantiate these early findings by measuring reflex amplitudes across longer latency periods and exploring the variability of reflexes within each subject. Methods: Forty-eight subjects with and without chronic ankle instability (CAI) walked on a treadmill at 4 km/h while activity of the lateral gastrocnemius (LG) was measured via surface electromyography. Non-noxious stimulations were elicited randomly to the ipsilateral sural nerve at the mid-stance phase of gait, and reflex amplitudes were calculated offline by comparing muscle activity during unstimulated and stimulated gait cycles. Two primary outcome measures were compared between groups at the middle latency (MLR: 80–120 ms) and late latency (LLR: 120–150 ms) time windows: (1) average reflex amplitudes and (2) standard deviation of reflex amplitudes for each subject across 10 trials. Results: Both groups demonstrated an equal amount of LG inhibition at the MLR and LG facilitation at the LLR. However, subjects with CAI showed significantly higher variability in LLR amplitude across trials than healthy controls. Conclusions: Increased variability of cutaneous reflex amplitudes may relate to symptoms associated with CAI. These findings suggest that reflex variability following sural nerve stimulation could serve as an objective measure to track treatment progress in patients with CAI, offering clinicians a new tool for conducting rehabilitation assessments in a controlled environment.

## 1. Introduction

Lateral ankle sprains (LASs) are the most common lower limb injuries in both athletic and general populations alike [1]. Beyond initial recovery, an estimated 40–70% of individuals experiencing LASs develop long-term deficits, which comprise chronic ankle instability (CAI) [2,3]. This condition is characterized by a variety of symptoms, including deficient postural control and proprioception, perceptions of ankle instability, and recurrent LASs [2,3]. CAI has a significant burden on public health, as those with the condition are at a higher risk of developing early onset osteoarthritis and often report diminished general function of the ankle, lower levels of physical activity, and kinesiophobia, which subsequently contribute to lower health-related quality of life measures [4,5].

One of the emerging topics of CAI research is the contribution of cutaneous reflexes, a polysynaptic neuromuscular pathway that serves to generate an appropriate compensatory motor output in response to tactile sensation. Modulation of this sensory-motor response is dependent on the phase of movement, intensity of perturbation, or innervation area stimulated. However, the goal of this modulation will be to maintain the current motor program and prevent loss of postural control or injury [6]. In healthy individuals, cutaneous reflexes assist in maintaining a normal gait pattern in the presence of an obstacle [6,7,8,9,10]. For example, during the stance phase, stimulation of the sural nerve results in the inhibition of the gastrocnemius as a protective unloading response to allow the transfer of weight from one limb to the other in the event of an unavoidable perturbation [10]. In those with CAI, the current literature has identified alterations of these reflex patterns during both static and dynamic activities, indicating changes at the spinal level following an LAS [11,12,13,14]. Specifically, Madsen et al. [14] identified a lack of gastrocnemius inhibition in the middle latency during the stance phase of gait in subjects with CAI, suggesting a lack of the unloading response used to maintain postural stability which, as a result, may contribute to higher injury risk.

Middle latency reflexes (MLRs), occurring ~80–120 ms post-stimulation in the lower limb, are representative of reflex modulation via interneurons at the spinal level. However, reflexes occurring at longer latencies (~120–150 ms) may also be important in understanding the entire motor response following perturbation. Like MLRs, long latency reflexes (LLRs) are context dependent, modulating corrective motor responses based on the goals of the task and position of the body while integrating all sensory information including visual feedback [15]. Unlike MLRs, which modulate motor output in the spinal cord to generate specific joint motions, LLRs are thought to be influenced by cortical processing, which contributes to the systemic response to maintain postural control during functional activities [15,16]. Aside from sensory–perceptual deficits at the spinal level, cortical contributions to reflexive alterations seen among this group may indicate changes as a result of learned behaviors or perceptions from previous experience [15]. A recent study found cerebral activity among those with CAI deviated from healthy controls, which may contribute to abnormal pain processing and neuromuscular strategies [17]. Considering those with CAI commonly experience feelings of instability, pain, and diminished self-reported function well after initial tissue healing, an exploration of LLRs may allow for a more thorough understanding of the contributive sources of reflex alterations over the course of recovery from LASs or throughout rehabilitation. Furthermore, in identifying the primary sources of sensorimotor changes seen in those with CAI, these sources may serve as potential biomarkers for neuromuscular recovery, allowing for more impairment-based treatment plans.

While other studies exploring reflexes in CAI subjects have focused on alterations in specific patterns or average muscle activity following cutaneous stimulation during a particular task, recent research in our lab provides evidence that these alterations, namely, in the lateral gastrocnemius (LG), may be due to variability in motor output, resulting in a general “lack” of significant reflexes reported among CAI subjects [14,18]. Considering the protective role cutaneous reflexes play in dynamic activity, inconsistency in motor output across perturbations may also contribute to the perceived instability seen in this population, as reflexive activity is intermittently misaligned with the muscle activity required to continue a smooth movement cycle and avoid stumbling [18]. Additionally, the stance phase of gait is of particular interest, as previous findings indicate abnormal reflexes among those with CAI during both standing [12] and drop landing tasks [13]. Inappropriate corrective responses from the stance limb, stemming from either spinal or supraspinal origins, would leave those with CAI at greater risk of stumbling and potential injury. Therefore, the purpose of this study was to observe middle and late latency cutaneous reflex patterns of the LG during mid-stance of gait in healthy individuals and those with CAI.

## 2. Materials and Methods

### 2.1. Participants

This study is a specific exploration of data collected from two larger studies that measured reflex patterns of 4 lower leg muscles throughout the gait cycle [18,19]. Forty-eight physically active adults, defined as participating in ≥120 min of physical activity participation per week, volunteered for this study. All subjects were neurologically intact (no neuromuscular conditions, such as Parkinson’s or multiple sclerosis) and had no history of fractures, surgeries in the lower extremity, or acute injury to the lower extremity within 6 weeks prior to data collection. Subjects were recruited into either the CAI group or control group using standard inclusion criteria endorsed by the International Ankle Consortium [20]. Specifically, all subjects completed the Identification of Functional Ankle Instability (IdFAI) questionnaire. Those with a history of at least 1 acute LAS and an IdFAI score of ≥11 in one limb were placed in the CAI group, while subjects with no history of LASs who scored 0 on both limbs were placed in the control group. The first LAS sustained by the CAI subjects was at least 1 year prior to enrollment and caused inflammatory symptoms that resulted in one or more interrupted days of desired physical activity. No CAI subject had sustained an LAS within 3 months of data collection. Table 1 provides pertinent group demographics along with average IdFAI scores and number of ankle sprains.

### 2.2. Electromyography

For all subjects, muscle activity of the LG was measured via either wired Delsys Bagnoli bipolar electrodes (Delsys Inc., Natick, MA, USA) or wireless disposable Ag/AgCL surface electrodes (Biopac Systems, Inc., Goleta, CA, USA). Each subject was asked to isometrically plantarflex against resistance applied by the researcher to identify the LG muscle belly. An area of approximately 2–3 inches in diameter was cleansed and the electrodes were applied and secured with clear medical tape. For subjects undergoing the wireless application, 2 electrodes were applied approximately 2 cm apart over the muscle belly, ensuring no contact between electrodes measuring adjacent muscles. A disposable grounding electrode was also placed over the tibial tuberosity of the test limb. For subjects undergoing the wired application, a single bipolar electrode was placed over the muscle belly and secured with both medical tape over the electrodes themselves and self-adhering tape around the lower leg. A metal grounding electrode with conducting gel was also placed over the acromion process ipsilateral to the test limb. Electrical leads of the wireless electrodes were connected and secured with medical tape over the electrode and through loops in the leads to limit movement artifacts. Leads were then connected to wireless transmitters which communicated with a Biopac MP160 recording system with EMG100c amplifiers. Wired electrode leads were looped and taped, connected to an 8-channel Delsys Bagnoli input module secured to a belt around the subject’s hips and then to a 16-channel Delsys amplifier. This amplifier communicated with a Biopac MP160 acquisition system to collect EMG data at a sampling rate of 2000 Hz.

### 2.3. Electrical Stimulation

A stimulating bar electrode (Ambu, Inc., Columbia, MD, USA) with conducting gel was affixed over the sural nerve just posterior to the lateral malleolus on the stimulated limb. The test limb was determined as the most affected limb in CAI subjects and was matched in the control group based on limb dominance to ensure that the same number of dominant and non-dominant limbs were measured in each group. A DS7A constant current stimulator (Digitimer North America, LLC, Ft. Lauderdale, FL, USA) connected to a custom-built latency device was used to administer 5-train pulse stimulations. The perceptual threshold (PT) was first identified by increasing the stimulation amplitude from zero until the subject reported any sensation from the electrical impulse around the foot or ankle. The radiating threshold (RT) was then identified by increasing the amplitude until the area of sensation grew into the lateral foot and up into the lower leg. The subject was asked to report when the sensation no longer grew in area, only in intensity. The final stimulation intensity for testing was this intensity (RT) multiplied by 2.5, which was then reduced in some subjects to ensure the stimulation was non-noxious and did not produce a withdrawal reflex. Prior to beginning the experimental protocol, the researcher elicited several test stimulations to confirm the intensity level for each subject met these criteria.

### 2.4. Protocol

**Figure 1:**
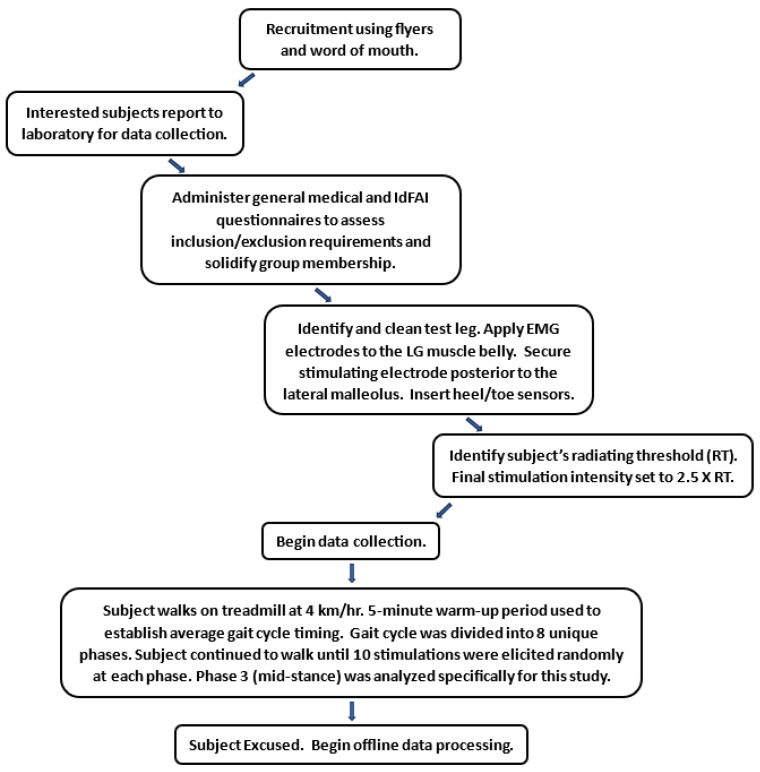
Flowchart outlining the data collection protocol for each subject.

All subjects walked on a treadmill at 4 km/h throughout the study protocol. During a 5 min warm-up period, the average gait cycle timing was identified for each subject, which was used to elicit stimulations during the mid-stance of the gait cycle. Heel–toe sensors inserted into both shoes collected heel strike data used for the timing of each stimulation, which was manually entered into a custom-built device. When manually triggering this device, a heel strike of the stimulated limb would elicit a stimulation at the entered latency corresponding with a particular phase of gait. As part of the larger studies, stimulation trials were randomized to ensure 10 stimulations occurred across 8 unique phases of the gait cycle (stance and swing), resulting in approximately 80 total stimulations throughout the walking task. The data analyzed in this study were taken solely from the 10 random stimulations elicited at the mid-stance (phase 3) of the gait cycle. All EMG, stimulation, and heel strike data were recorded using a Biopac MP160 acquisition system and Acqknowledge 5.0 software (Biopac Systems, Inc., Goleta, CA, USA). Figure 1 provides a flowchart that outlines the data collection protocol for each subject.

### 2.5. Data Processing

All data processing was completed using AcqKnowledge 5.0 software (Biopac Systems, Inc., Goleta, CA, USA). Raw EMG data were filtered at a low-frequency cutoff of 50 Hz and a high-frequency cutoff of 500 Hz. The root mean square (RMS) was then derived for the smoothed signals for the LG. Stimulated trials labeled by phase during testing were reviewed for step timing consistency, leaving approximately 8–10 trials for all subjects. Unstimulated gait cycles were labeled accordingly, which were all at least 3 gait cycles before or after a stimulated trial. This left approximately 200 unstimulated trials for each subject, which were then ensemble averaged for comparison to stimulated trials.

To compare values between subjects, all waveforms were normalized as a percentage of each muscle’s maximum EMG amplitude for each subject during unstimulated trials (%MVC). Unstimulated waveforms of all muscles were aligned at the point of the triggering heel strike voltage using data from heel–toe sensors during phase 1 stimulations. To explore both the MLR and LLR, mean values from the unstimulated ensemble average and individual stimulated waveforms were extracted at 80–120 ms and 120–150 ms post-stimulation, respectively. These time windows were chosen because they preceded any voluntary contraction and they matched the previously published cutaneous reflex literature [8,10,13]. Stimulated waveforms were visually inspected to confirm that this window accurately captured the MLR and LLR for each subject. Average unstimulated reflex values were then subtracted from each stimulated value for each subject to acquire the final reflex amplitudes in %MVC for each trial. Additionally, normalized unstimulated LG amplitudes during these latencies were extracted for analysis of background muscle activity.

### 2.6. Statistical Analysis

Three separate two-way mixed-factor ANOVAs (one for each dependent variable) were conducted to determine whether there was an interaction between the dependent variable and two independent variables. The independent variables remained consistent for each analysis and included a between-subjects factor (group) at two levels (CAI and control) and a within-subjects factor (latency) at two levels (middle latency and long latency). The dependent variable for the first analysis was the background EMG activity of the LG, the dependent variable for the second analysis was the average LG reflex amplitude, and the dependent variable for the third analysis was the standard deviation of the LG reflexes across 10 stimulation trials for each subject. For each ANOVA, we checked necessary assumptions including assessing for significant outliers, whether the dependent variable was normally distributed, if the variance of the dependent variable was equal between groups of the between-subjects factor, and if there was homogeneity of covariances.

## 3. Results

### 3.1. Background EMG

There were no outliers in the data as assessed by the inspection of boxplots. Histograms showed that the background LG activity was normally distributed for each combination of the levels of the between- and within-subjects factors. Studentized residuals were also evaluated to determine whether any observed values of LG BEMG were significantly different (more than ±3 standard deviations) from its predicted variable. There was one outlier present in the CAI group, which had a studentized residual value of −3.27. This variable represented a genuinely unusual value (not a data entry value) and was left in the final analysis since the results of the ANOVA did not change whether this subject was included or excluded. The residuals of LG BEMG were found to be normally distributed at the middle and long latencies, as assessed by normal Q-Q plots. Finally, we found there was homogeneity of variances (*p* > 0.05) and covariances (*p* = 0.619) by assessing Levene’s test and Box’s test, respectively. There was no statistically significant interaction between the group and latency on LG BEMG, F(1,46) = 0.794, *p* = 0.378, and partial ŋ^2^ = 0.017. The main effect of latency showed a statistically significant difference in mean LG BEMG at the different latency points, F(1,46) = 31.8, *p* < 0.001, and partial ŋ^2^ = 0.017, with the long latency time window having an average of 11.5 ± 2.05% (95% CI = 7.4 to 15.7) more LG activity compared to the middle latency window. The main effect of the group showed no statistical difference in mean LG activity between groups regardless of the latency time window, F(1,46) = 2.15, *p* = 0.149, and partial ŋ^2^ = 0.045, with the control group having an average LG activity of 63.83 ± 2.6% (95% CI = 58.7 to 69.1) and the CAI group averaging 69.3 ± 2.60% (95% CI = 64.0 to 74.5).

### 3.2. Average LG Reflex Amplitudes

**Figure 2:**
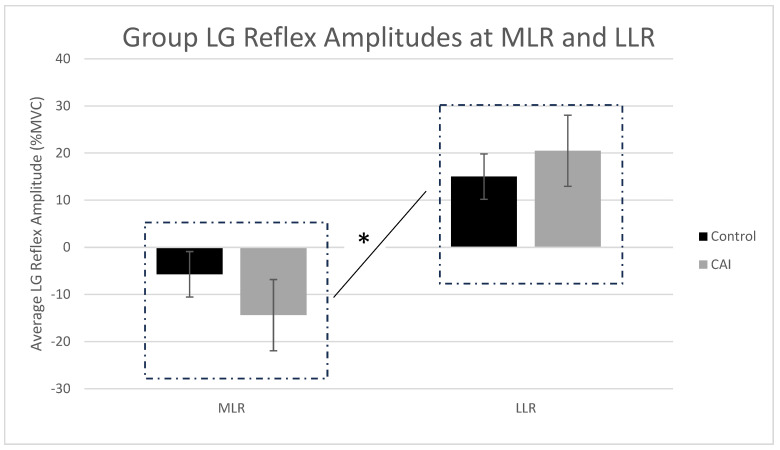
Average (±SE) group reflex amplitudes for the LG at the middle (80–120 ms post-stimulation) and long (120–150 ms post-stimulation) latencies. Data are presented as a percentage of the subjects’ maximum voluntary contraction of the LG across unstimulated gait cycles. The asterisk (*) shows the significant main effect of latency, whereby subjects (regardless of group) demonstrated significantly different LG reflex amplitudes between the middle and long latencies.

There were no outliers present in the data, and the average LG reflex amplitudes at each latency window were normally distributed for both groups. The studentized residuals were all within ±3 SDs, indicating no outliers, while the normal Q-Q plots of studentized residuals revealed a normal distribution. The homogeneity of variances was confirmed via Levene’s test (*p* > 0.05) at both latencies. Box’s test revealed that the observed covariance matrices of the dependent variables were not equal between groups (*p* = 0.026). Tests of within-subjects effects found that there was no statistically significant interaction between group and latency for the mean LG reflex amplitudes, F(1,46) = 2.51, *p* = 0.12, and partial ŋ^2^ = 0.052. Figure 2 shows average reflex amplitudes for each group at both MLR and LLR reflex latencies. The main effect of latency showed a statistically significant difference in mean LG reflex amplitudes between the MLR and LLR time points, F(1,46) = 38.7, *p* = 0.001, partial ŋ^2^ = 0.457, and Cohen’s d = 0.87, with the MLR reflex indicating an average LG inhibition of –10.0 ± 3.41% (95% CI = −16.9 to −3.2) and the LLR showing an average LG facilitation of 17.8 ± 5.35% (95% CI = 7.0 to 28.5). The main effect of the group showed that the average LG reflex amplitude was statistically similar between groups regardless of latency, F(1,46) = 0.042, *p* = 0.839, and partial ŋ^2^ = 0.001.

### 3.3. Subject Variability of LG Reflex Amplitudes

**Figure 3:**
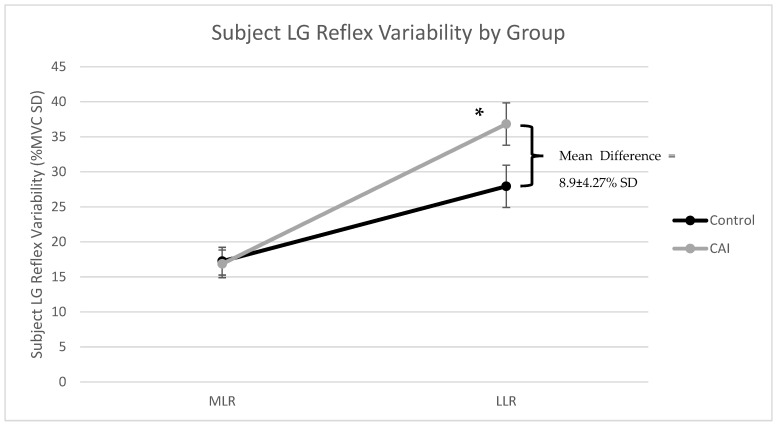
Cutaneous reflex variability was determined for each subject by calculating their standard deviation of LG reflex amplitudes across all stimulation trails. This graph shows the average (±SE) reflex variability for the CAI and control subjects at the MLR and LLR. The asterisk (*) indicates a statistically significant main effect for the group at the LLR.

The standard deviations of LG amplitudes across 10 stimulation trials that were calculated for each subject were first reviewed for outliers and evidence of a normal distribution. No outliers were identified following a review of boxplots. Both groups were found to be normally distributed at the long latency time window (*p* > 0.05). However, the results of the Shapiro–Wilk test of normality found that the control group (*p* = 0.018) and the CAI group (*p* = 0.002) were not normally distributed at middle latency. Despite this Shapiro–Wilk test violation, the ANOVA analysis was still considered appropriate because both groups were similarly skewed at this middle latency. Specifically, the Z-scores for skewness and kurtosis were 2.07 for the control group and 2.21 for the CAI, which fall within a Z-score significance level of 0.01. The studentized residuals were all within ±3 SDs indicating no outliers, while the normal Q-Q plots of studentized residuals revealed a normal distribution. The homogeneity of variances was confirmed via Levene’s test (*p* > 0.05) at both latencies. Box’s test revealed that the observed covariance matrices of the dependent variables were equal between groups (*p* = 0.209). Tests of within-subjects effects found that there was a statistically significant interaction between the group and latency for LG reflex variability, F(1,46) = 4.79, *p* = 0.034, and partial ŋ^2^ = 0.094. The simple effect of latency found that there was a statistically significant effect of latency on reflex variability for both the control, F(1,23)= 14.9, *p* < 0.001, and partial ŋ^2^ = 0.393, and the CAI group, F(1,23) = 38.5, *p* < 0.001, and partial ŋ^2^ = 0.626. Both groups experienced significantly more reflex variability during long latency compared to middle latency, with the control group experiencing a mean difference of 10.7 ± 2.77% SD and the CAI group experiencing a mean difference of 19.9 ± 3.22% SD. The simple effect for the group found that control and CAI subjects had similar reflex variability at middle latency, F(1,46) = 0.018, *p* = 0.893, and partial ŋ^2^ = 0.000, but there was a statistically significant difference in reflex variability at long latency between groups, F(1,46) = 4.35, *p* = 0.043, partial ŋ^2^ = 0.086, and Cohen’s d = 0.6. On average, the CAI subjects experienced 8.90 ± 4.27% (95% CI = 0.3 to 17.5) more reflex variability at long latency compared to controls. Figure 3 shows the subject variability of LG reflex amplitudes for each group at both middle and long latencies.

## 4. Discussion

Our first analysis was conducted to determine whether background LG activity (extracted from the unstimulated gait cycles) was different between groups. According to the theory of automatic gain compensation, human reflex amplitudes, whether facilitatory or inhibitory, increase proportionately with the amount of voluntary muscle activity at the time of perturbation. This phenomenon likely occurs because stronger muscle contraction causes an increase in motoneuron pool excitability [21,22,23]. Therefore, if one of our groups had significantly more LG activity across the stance phase, automatic gain compensation may cause their cutaneous reflex amplitudes to be more pronounced. Our results found that, on average, the LG was more active across the long latency time window compared to the middle latency. This result is to be expected since the LG becomes more active as a person moves closer to the push-off phase of the gait cycle. However, this increase in LG activity from the MLR to the LLR was present in both the control and CAI subjects, and we found no statistical difference in background muscle activity between groups at either time point. Thus, any subsequent group differences in reflexes following sural nerve stimulation could be interpreted as variations in motor control mechanisms (e.g., spinal and supraspinal pathways mediating excitatory and inhibitory reflexes) rather than merely the result of differences in background LG activity during stance.

The reflex amplitudes at the MLR and LLR were also statistically similar between groups. Specifically, stimulation of the sural nerve at midstance resulted in sufficient inhibition of the LG at the MLR followed by an abrupt facilitation at the LLR. With respect to the MLR inhibition, this motor control response is to be expected in the gastrocnemius among healthy, neurologically intact adults, as the inhibition helps prepare the stance limb to unload and shift weight to the contralateral limb should the perturbation result in a stumble [10]. Surprisingly, our results did not match those from a previous study reported by Madsen et al. [14] that found people with CAI failed to reach a statistically significant LG inhibition at midstance, an abnormal reflex pattern that may account for feelings of instability and/or increased injury risk among this cohort. The exact cause for this discrepancy between studies is unknown, but we suspect the facilitation of the LG at the LLR window may be involved. Although not statistically different, the CAI group had the highest average LG facilitation at the LLR. Additionally, the subjects with CAI demonstrated the most variability in LLR amplitudes across 10 trials. Perhaps the small sample size of subjects measured in the 2019 study experienced this fluctuating LG facilitation within the later stages of the MLR (around 100–120 ms), effectively nullifying any inhibition of the muscle that may have been present at an earlier latency. More research is certainly needed to help substantiate MLR amplitudes in the LG among people with CAI, but current evidence suggests variability of reflex amplitudes across several stimulation trials may contribute to the inconsistent group averages reported in the CAI literature.

The reflex amplitudes measured at the LLR are worthy of further discussion, as no previous study has investigated this cutaneous reflex output in people with CAI. Our results found that, on average, both groups transitioned from the LG inhibition at the MLR to a sharp facilitation at the LLR. Previous research has found similar phasic cutaneous reflexes in men. Specifically, a study by Jenner and Stephens [24] measured cutaneous reflexes in the first dorsal interosseous muscle of the hand following stimulation to the digital nerves of the index finger as subjects maintained a steady isometric contraction. They found a triphasic reflex consisting of a short latency excitation, short latency inhibition, and finally, a long latency excitation. Interestingly, the long latency excitation of this cutaneous reflex was reduced in patients with dorsal column lesions and even absent in patients with damage to the motor cortex. Thus, the researchers concluded that the shorter latency excitation and inhibition components of the cutaneous reflex are mediated primarily by spinal pathways, while the long latency excitatory component is of supraspinal origin. The afferent impulses presumably transmit through dorsal columns to the sensorimotor cortex and descend to the lower motoneuron pool via the corticospinal tract.

From a lower extremity perspective, there are confirmed connections between corticospinal tract neurons and both the tibialis anterior and the soleus motoneurons for medium latency (MLR) and long latency (LLR) reflexes. Petersen et al. [25] and Christensen et al. [26] investigated the time course of corticospinal input to the different reflex components (SLR, MLR, and LLR) in the TA. Whereas the facilitatory effects produced with corticospinal input for the SLR and MLR time intervals were negligible, facilitation was reported for the LLR. In both studies, it was concluded that a transcortical reflex pathway contributes to the generation of the LLR in this muscle. Similarly, Sinkjaer et al. [27] suggested a transcortical mediated loop at the LLR latency for the soleus muscle. Pertinent to the current results, the cortical influence for the soleus was apparent at much longer latencies (>114 ms) than that reported for the tibialis anterior muscle, and this coincides with our identification of the LLR at 120–150 ms for the lateral gastrocnemius muscle via cutaneous stimulus. Taub et al. [28] also confirmed that after 85 ms, the muscular response during perturbation is influenced by cortical structures via direct monosynaptic projections. Thus, consistent with the literature for the tibialis anterior muscle when walking [26], cortical input via a transcortical reflex loop may play an important role for the soleus muscle as well. Our study was focused on the lateral gastrocnemius muscle (a muscle with a similar function to the soleus), and it remains to be determined whether cortical input directly affects this motor pool when walking. However, the evidence from both the TA and soleus suggests that cortical input is driving the longer latency reflexes observed from muscle perturbation.

Perhaps it is the increased LLR facilitation, coupled with the increased variability, presumably from transcortical pathways, that contributes in part to CAI. Previous research has reported that there is generally greater variability in LLRs when compared to the MLR and SLR reflexes (see Figure 6, Taube et al. [28]). We observed the same increased variability (LLR variability > MLR variability) with both control and CAI subjects. However, pertinent to our data, there was significantly greater variability for the LLR in the CAI group. This excessive variability may in fact be a driving force in both the physiology and perception of ankle instability. People with CAI experience variable symptoms, meaning they don’t always feel unstable or have episodes of giving way [18]. The heightened variability in the LLR in the CAI group may contribute to this perception and provide an avenue to explore with CAI patients. Support for the idea that variability in neurophysiological function relates to behavioral perception does appear in the literature [29]. For example, impaired ankle inversion proprioception among the elderly has been correlated with fear of falling inventories (r = −0.61), with individuals with higher fear of falling scores being associated with lower proprioception scores [29]. It remains to be seen whether this type of association holds for CAI patients.

### 4.1. Limitations

There were several limitations in this study. First, we had to use a treadmill during data collection to ensure consistency in muscle activity across hundreds of gait cycles. Unfortunately, the treadmill may not simulate a natural gait for some subjects, making it difficult to assume that these findings will persist as people walk in a natural environment. Second, our sample size, although larger compared to previous cutaneous reflex reports, is limited, and may affect statistical validity. Finally, we did not employ a questionnaire to measure functional limitations across our subject pool, which makes it challenging to generalize our findings across all deficits associated with CAI.

### 4.2. Future Clinical Relevance

The long-term consequences and reduced quality of life in individuals with CAI create a burden on the healthcare system [4]. The burden could be relieved with more objective markers that could be targeted in the assessment and rehabilitation. Including cutaneous reflexes as a consistent part of the ankle assessment might glean a more holistic understanding of neuromuscular deficiencies that are present. Assessment of cutaneous reflexes is one such opportunity to provide a clinical marker of ankle instability. It could serve as both an assessment tool once the complaints of instability and giving way have been reported and, more importantly, it could be monitored throughout the rehabilitation process to determine the types of inventions that are beneficial to this population. Additionally, monitoring reflexes throughout the rehabilitation process can assist in determining the efficacy of current rehabilitation protocols and when a return to activity should be recommended. An additional challenge in treating individuals with CAI is the intermittent symptoms reported by this population. If abnormal cutaneous reflexes continue to be identified in individuals with CAI, then clinicians can measure reflex variability across the MLR and LLR as an objective measure of those symptoms. This information, in addition to the patient-reported outcome measures currently used by clinicians, could create yet another opportunity for clinicians to target treatment progression. Understanding cutaneous reflex variability could assist in understanding the intermittent symptoms of patients with CAI and provide another objective assessment of the ankle evaluation that can guide clinical decision making.

## 5. Conclusions

This study found that people with a history of ankle sprains and residual feelings of instability have significantly higher variability in LLR amplitudes across stimulation trials compared to healthy controls. This finding suggests that reflex variability following non-noxious sural nerve stimulation may relate to symptoms associated with CAI and could serve as an objective measure to track treatment progress in a controlled clinical environment.

## Figures and Tables

**Table 1:** Average (±SD) subject demographics by group.

	Control (*n* = 24)	CAI (*n* = 24)
Sex	7 M, 17 F	8 M, 16 F
Age (yrs.)	20.2 ± 1.98	19.9 ± 1.57
Height (m)	1.67 ± 0.09	1.68 ± 0.1
Weight (kg)	64.0 ± 9.48	65.7 ± 16.19
IdFAI Scores	0	17.3 ± 3.8
# of Ankle Sprains	0	2.1 ± 1.07

## Data Availability

The raw data supporting the conclusions of this article will be made available by the authors upon request.
